# Polymeric Caffeic Acid Acts as a Nasal Vaccine Formulation against *Streptococcus pneumoniae* Infections in Mice

**DOI:** 10.3390/pharmaceutics13040585

**Published:** 2021-04-20

**Authors:** Rui Tada, Hidehiko Suzuki, Miki Ogasawara, Daisuke Yamanaka, Yoshiyuki Adachi, Jun Kunisawa, Yoichi Negishi

**Affiliations:** 1 Department of Drug Delivery and Molecular Biopharmaceutics, School of Pharmacy, Tokyo University of Pharmacy and Life Sciences, 1432-1 Horinouchi, Hachioji, Tokyo 192-0392, Japan; y134055@toyaku.ac.jp (M.O.); negishi@toyaku.ac.jp (Y.N.); 2 Laboratory of Vaccine Materials and Laboratory of Gut Environmental System, National Institutes of Biomedical Innovation, Health and Nutrition (NIBIOHN), 7-6-8 Saito-Asagi, Ibaraki, Osaka 567-0085, Japan; hide-suzuki@nibiohn.go.jp (H.S.); kunisawa@nibiohn.go.jp (J.K.); 3 Laboratory for Immunopharmacology of Microbial Products, School of Pharmacy, Tokyo University of Pharmacy and Life Sciences, 1432-1 Horinouchi, Hachioji, Tokyo 192-0392, Japan; ymnkd@toyaku.ac.jp (D.Y.); adachiyo@toyaku.ac.jp (Y.A.); 4 Division of Mucosal Immunology and International Research and Development Center for Mucosal Vaccines, Department of Microbiology and Immunology, The Institute of Medical Science, The University of Tokyo, 4-6-1 Shirokanedai, Minato-ku, Tokyo 108-8639, Japan

**Keywords:** polyphenol, mucosal vaccine, mucosal adjuvant, PspA, *Streptococcus pneumoniae*

## Abstract

Infectious diseases are the second leading cause of death worldwide, highlighting the importance of the development of a novel and improved strategy for fighting pathogenic microbes. *Streptococcus pneumoniae* is a highly pathogenic bacteria that causes pneumonia with high mortality rates, especially in children and elderly individuals. To solve these issues, a mucosal vaccine system would be the best solution for the prevention and treatment of these diseases. We have recently reported that enzymatically polymerized caffeic acid (pCA) acts as a mucosal adjuvant when co-administered with antigenic proteins via the nasal route. Moreover, the sources of caffeic acid and horseradish peroxidase are ingredients found commonly in coffee beans and horseradish, respectively. In this study, we aimed to develop a pneumococcal nasal vaccine comprising pneumococcal surface protein A (PspA) and pCA as the mucosal adjuvant. Intranasal immunization with PspA and pCA induced the production of PspA-specific antibody responses in the mucosal and systemic compartments. Furthermore, the protective effects were tested in a murine model of *S. pneumoniae* infection. Intranasal vaccination conferred antigen-dependent protective immunity against a lethal infection of *S. pneumoniae*. In conclusion, pCA is useful as a serotype-independent universal nasal pneumococcal vaccine formulation.

## 1. Introduction

Although there have been substantial advances in modern medicine, infectious diseases are the second leading cause of death worldwide [1]. Hence, there is an urgent need to develop novel drugs or strategies for the prevention and treatment of infectious diseases. For this purpose, vaccines are considered an important path to overcome infectious diseases and are being actively researched. However, there are only about 20 infectious diseases that can be prevented by a vaccine, termed vaccine-preventable diseases (VPD) [2].

*Streptococcus pneumoniae* is a highly pathogenic bacteria that causes pneumonia, meningitis, and septicemia with high mortality rates, especially in children and elderly individuals. Moreover, the increase in the number of antibiotic-resistant *S. pneumoniae* strains has made the treatment and management of pneumococcal infections more challenging [3]. Although the 23-valent pneumococcal polysaccharide vaccine (PPSV-23) and the 13-valent pneumococcal conjugate vaccine (PCV-13) are currently licensed and clinically applied as vaccines [4], recent evidence indicates that the diseases caused by pneumococcal infections are responsible for 3–5 million deaths annually [5].

The major drawbacks of existing pneumococcal vaccines are as follows. (1) The protective effects induced by administering these vaccines are serotype dependent. This is because these vaccines use the capsular polysaccharide, located at the outermost layer of *S. pneumoniae*, as the antigen, which varies among serotypes [6,7]. Additionally, in recent years, there has been an increase in the prevalence of serologically non-typeable *S. pneumoniae* (also known as non-encapsulated *S. pneumoniae* due to the lack of a capsule); these vaccines are ineffective against such strains [8]. (2) Since existing pneumococcal vaccines are administered systemically (e.g., intramuscular or subcutaneous injections), antigen-specific immunoglobulin (Ig) G is induced in the blood, but not in the upper respiratory tract (including the nasal mucosa), which is the site of infection and/or colonization [9]. (3) Polysaccharide antigens inadequately exert long-lasting immune memory responses [10]. Furthermore, children and elderly individuals, the major targets for pneumococcal vaccines, inherently respond poorly to polysaccharide antigens because of the lack of T cell memory [11]. To overcome these issues, the development of a vaccine system based on an antigenic protein that is expressed in a broad range of *S. pneumoniae* strains and is serotype independent is crucial. Moreover, developing a mucosal vaccine system that effectively elicits an immune response in the upper respiratory tract is also important.

Pneumococcal surface protein A (PspA) is expressed on the cell surface of all *S. pneumoniae* strains isolated to date. PspA is classified into three families and six clades (family 1, clades 1 and 2; family 2, clades 3–5; family 3, clade 6) [12,13]. Although the sequence varies between the *S. pneumoniae* strains, the PspA-specific immune response is known to confer serotype-independent protection against pneumococcal infection [14]. Notably, the PspA protein derived from Rx1 (family 1, clade 2) induces potent cross-reactivity against PspA family 1 and 2 [15,16]. Hence, the protein is a promising antigen candidate for use in a universal pneumococcal vaccine system. Moreover, *S. pneumoniae* is transmitted through the upper respiratory tract, including the nasal mucosa. Therefore, such mucosal vaccine systems are ideal and act by inducing antigen-specific immune responses in the target region. Although mucosal vaccines are crucial for the prevention and treatment of various infectious diseases, very few are readily available in clinics. Protein antigens have intrinsically poor immunogenicity when administered through the mucosal route; thus, an appropriate adjuvant is required to induce mucosal antigen-specific immune responses. Nevertheless, there is currently a need for the further development of safe and effective mucosal adjuvants [17,18,19,20].

In our previous studies, we enzymatically synthesized polymerized polyphenols (such as caffeic acid [CA]) from phenylpropanoids using horseradish peroxidase (HRP) [21,22,23,24]. We had reported that polymerized CA (pCA) acts as a mucosal adjuvant when co-administered with antigenic proteins via the nasal route. This method resulted in the induction of a higher titer of antigen-specific mucosal and systemic antibody responses in mice. Since intranasal administration of pCA does not exert any toxicity in mice, pCA maybe useful to the development of a safe mucosal vaccine system [25,26]. Further, the sources of CA and HRP are coffee beans and horseradish, respectively, which are easily available. Hence, we expect pCA to be useful as a highly safe mucosal adjuvant for nasal pneumococcal vaccine systems. In this study, we have tested the protective effects of a pCA-based nasal pneumococcal vaccine system using PspA as an antigenic protein against pneumococcal infections.

## 2. Materials and Methods

### 2.1. Animals and Materials

Seven-week-old BALB/cCrSlc female mice were procured from Japan SLC (Shizuoka, Japan) and kept under specific pathogen-free conditions. All experimental protocols involving animals were pre-approved by the Tokyo University of Pharmacy and Life Sciences Committee for Laboratory Animal Experiments (P17–26, P18–71, and P19–58), as well as by the National Institutes of Biomedical Innovation, Health and Nutrition committee (DS25–3R8). Bacterial infection studies on mice were performed under anesthesia with isoflurane gas, and all efforts were strictly taken to minimize pain. Furthermore, 3-(3,4-Dihydroxyphenyl)-2-propenoic acid (CA) was purchased from Tokyo Chemical Industry Co., Ltd. (Tokyo, Japan), and HRP was obtained from Merck Millipore (Billerica, MA, USA).

### 2.2. Preparation of pCA

pCA was synthesized as previously reported [24]. CA was dissolved in 1 M NaOH and neutralized by adding phosphate-buffered saline (PBS) at a final concentration of 20 mg/mL containing HRP (0.1 mg/mL). Thereafter, H_2_O_2_ (1.5 mol equivalent to CA) was added to the reaction mixture with stirring at 25 °C for 3 h and boiled for 20 min to remove HRP. The mixture was then centrifuged, and the supernatant was dialyzed (molecular weight cutoff: 50,000 Da) against ultrapure water and then lyophilized to collect pCA. The sample was then dissolved in endotoxin-free PBS (FUJIFILM Wako Pure Chemical Corporation, Osaka, Japan) to prepare a stock solution (10 mg/mL) that was sterilized by filtration through 0.22-μm filter membranes (Osaka Chemical Co., Ltd., Osaka, Japan). The stock solution was stored at −20 °C until further use.

### 2.3. Preparation of PspA Expression Plasmid

To prepare the expression vector, the PspA gene fragment was amplified by polymerase chain reaction (PCR) from a pET16b-PspA template containing the pspa gene from *S. pneumoniae* Rx1 (PspA family 1 and clade 2) using KOD One DNA polymerase (Toyobo, Tokyo, Japan) [27,28]. The following primer pairs were used for amplification: forward primer: 5′-ATCATATCGAAGGTAGGCATATGGAAGAATCTCCCGTA-3′; reverse primer: 5′-TTTAAGCAGAGATTACCTATCTAGATTATTCTGGGGCTGGAGTTT-3′. After treatment with DpnI (Toyobo) as a destroying plasmid template, the obtained PspA PCR fragment was cloned into the expression vector pCold1 (Takara Bio Inc., Shiga, Japan), linearized by NdeI (Takara Bio Inc.) in combination with XbaI (Takara Bio Inc.), using a Seamless Ligation Cloning Extract cloning method [29,30]. After the SLiCE reaction, the sample was immediately transformed into the *Escherichia coli* DH5α strain (BioDynamics Laboratory Inc., Tokyo, Japan).

### 2.4. Purification of Recombinant PspA Protein

To produce the recombinant PspA protein (PspAp), the prepared expression plasmid, named as a pCold1-PspA plasmid, was transformed into an *E. coli* BL21 (DE3) strain (BioDynamics Laboratory Inc.). The transformed cells were pre-cultured overnight in 5 mL of LB medium containing ampicillin at 37 °C. Further, pre-cultured cells (2 mL) were inoculated into 200 mL of LB medium containing ampicillin at 37 °C to reach an optical density value of 0.4. The cultured *E. coli* cells were transferred onto ice for 30 min and then incubated at 15 °C for 30 min. After incubation, 0.5 mM of isopropyl-D-thiogalactopyranoside (IPTG; FUJIFILM Wako Pure Chemical Corporation) was added to the culture and left for 24 h to induce the expression of PspA. The cell pellets were re-suspended in a TALON equilibration buffer (pH 7.0) containing 5% glycerol, 5 mM 2-mercaptoethanol (2-ME), and an ethylenediaminetetraacetic acid-free protease inhibitor cocktail (Nacalai Tesque, Inc., Kyoto, Japan) and sonicated extensively on ice to disrupt the cells. After removing the insoluble fraction by centrifugation, the obtained supernatant was subjected to a column packed with TALON metal affinity resin (Takara Bio Inc.). The column was washed with TALON equilibration buffer (pH 7.0) containing 5% glycerol and 5 mM 2-ME, followed by another TALON equilibration buffer (pH 7.0) containing 5% glycerol, 5 mM 2-ME, and 10 mM imidazole to remove non-specific proteins bound to the resins. The bound recombinant PspAp was eluted with the final TALON equilibration buffer (pH 7.0) containing 5% glycerol, 5 mM 2-ME, and 150 mM imidazole. The eluted protein fractions were dialyzed against PBS using a dialysis membrane (molecular weight cutoff: 3500 Da). After dialysis, endotoxin removal was performed using Pierce^™^ High Capacity Endotoxin Removal Spin Columns (Thermo Fisher Scientific, Waltham, MA, USA) according to the manufacturer’s instructions. The concentration of purified PspA protein was quantified using the BCA Protein Assay Kit (FUJIFILM Wako Pure Chemical Corporation). The purity of PspAp was assessed by sodium dodecyl sulfate-polyacrylamide gel electrophoresis, followed by Coomassie brilliant blue staining (Figure S1). The purified PspA protein was stored at −80 °C until further use.

### 2.5. Immunization and Sampling Schedule for the Assessment of PspA-Specific Antibody Production

The mice were nasally immunized with PBS (vehicle), PspA alone (5 µg/mouse), pCA alone (100 µg/mouse), or PspA (5 µg/mouse) with pCA (100 µg/mouse) at a volume of 13 µL once a week for 3 consecutive weeks. To assess systemic PspA-specific antibody responses, the blood was collected and incubated at 25 °C for 30 min. Further, the obtained samples were incubated for 1 h at 4 °C, and the serum sample was collected after centrifugation at 1200× *g* for 30 min. The nasal wash (200 µL of cold D-PBS), vaginal wash (100 µL of cold D-PBS), and bronchoalveolar lavage fluid (1000 µL of cold D-PBS) were collected [16,31,32]. The samples were stored at −80 °C until use.

### 2.6. ELISA for Detection of PspA-Specific Antibody

A 96-well Sumilon ELISA H plate (Sumitomo Bakelite Co., Ltd., Tokyo, Japan) was coated with 50 ng of PspA in 0.1 M carbonate buffer (pH 9.5) overnight at 4 °C. After washing with PBS containing 0.05% Tween-20 (PBST), the wells were blocked with 1% bovine serum albumin (FUJIFILM Wako Pure Chemical Corporation) in PBST (BPBST) at 37 °C for 1 h. The plate was further washed and the samples was added to each well and incubated overnight at 4 °C. After washing with PBST, the plates were treated with peroxidase-conjugated anti-mouse IgA, IgG, IgG1, or IgG2a secondary antibodies (4000-fold dilution) (Southern Biotech, Birmingham, AL, USA) in BPBST, and color was developed using a tetramethylbenzidine substrate system (SeraCare Life Sciences, Inc., Milford, MA, USA). Color development was stopped by adding 1 N phosphoric acid, followed by the measurement of absorbance at 450 nm with 650 nm as a reference using a Synergy HTX Multi-Mode Microplate Reader (BioTek Instruments, Inc., Whiting, VT, USA) [33]. The endpoint titers were measured as the reciprocal of the last dilution, reaching a cut-off value set to twice the mean absorbance value of a negative control [34,35].

### 2.7. In vivo Pneumococcal Infection Study

The *S. pneumoniae* Xen10 strain (parental strain, A66.1 serotype 3), which expresses family 1, clades 1 and 2 PspA [36], (Caliper Life Sciences, Hopkinton, MA, USA), was cultured overnight in brain-heart infusion broth at 37 °C in a 5% CO_2_ atmosphere without aeration. Thereafter, the cultured *S. pneumoniae* cells were collected and the cells were washed twice and diluted with PBS. Based on immunization, the study involved four groups of animals receiving (1) vehicle (PBS), (2) PspA alone (5 µg/mouse), (3) pCA alone (100 µg/mouse), or (4) pCA (100 µg/mouse) in combination with PspA (5 µg/mouse) at a volume of 13 µL once a week for three consecutive weeks. Seven days after the final immunization, the mice were nasally challenged with 5.0 × 10^6^ colony-forming units (CFU) of *S. pneumoniae* Xen10 strain. The survival of mice was monitored for two weeks [27,28].

### 2.8. Statistical Analysis

Statistical analyses were performed by using the Kruskal–Wallis with Dunn’s post hoc test for antibody production or the Mantel–Cox test for survival assays, using GraphPad Prism 8 software (GraphPad Software, San Diego, CA, USA). Statistical significance was set at *p* < 0.05.

## 3. Results

### 3.1. Nasal Immunization of a PspA Antigen with pCA Elicits PspA-Specific Antibody Responses in the Mucosal and Systemic Compartments

**Figure 1:**
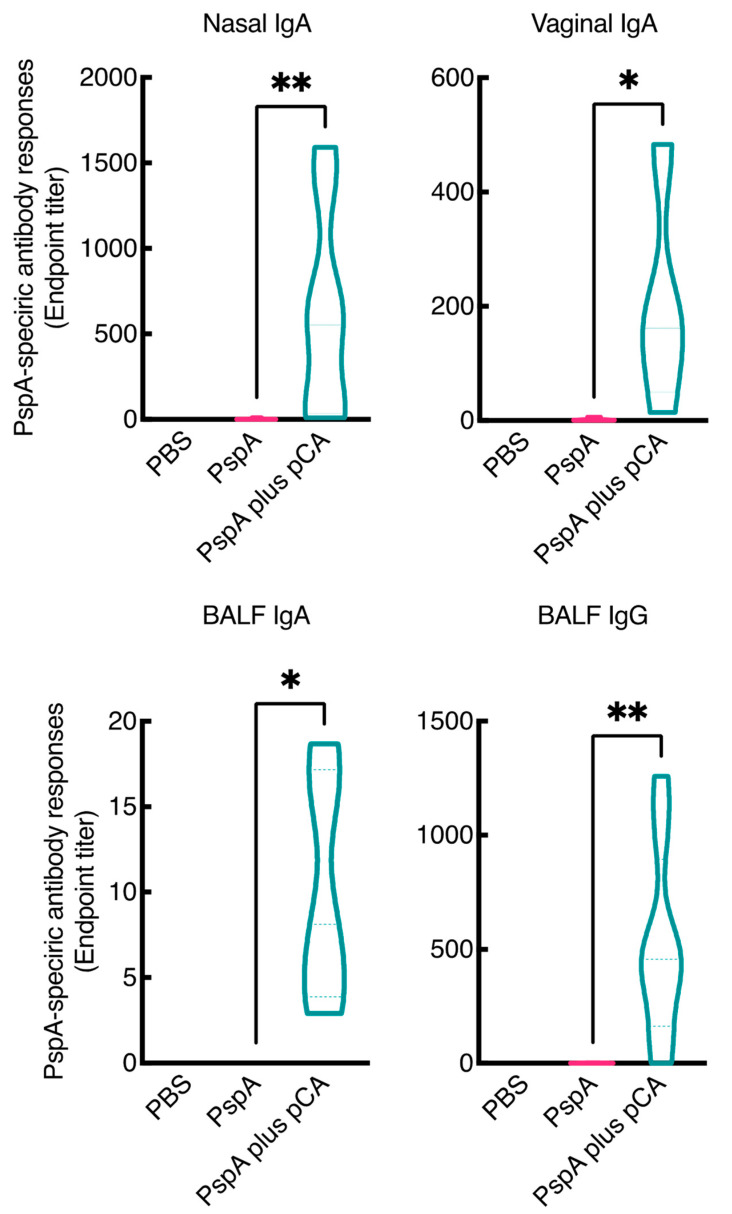
Induction of mucosal PspA-specific antibody responses in BALB/c female mice who nasally received PspA and pCA. BALB/c female mice were nasally immunized with vehicle (PBS), PspA (5 µg/mouse), or PspA (5 µg/mouse) in combination with polymeric caffeic acid (pCA) (100 µg/mouse) on days 0, 7, and 14. Seven days after the last immunization, the nasal wash, bronchoalveolar lavage fluid, and vaginal wash were collected. The PspA-specific antibodies in the samples were evaluated by enzyme-linked immunosorbent assay. Data are obtained from three independent experiments. Vehicle, *n* = 9; PspA, *n* = 12; PspA plus pCA, *n* = 12. Significant differences were calculated using the Kruskal–Wallis test with Dunn’s post hoc test. * *p* < 0.05, ** *p* < 0.01.

**Figure 2:**
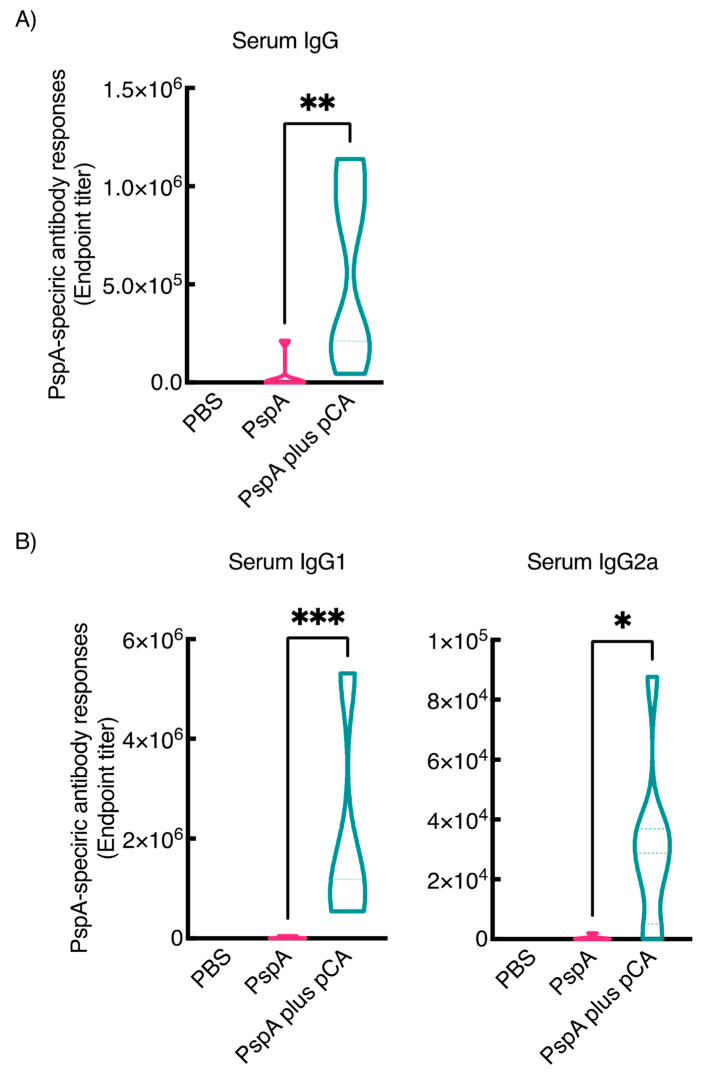
Induction of PspA-specific (**A**) serum total IgG responses and (**B**) serum IgG subclasses in BALB/c female mice that nasally received PspA and pCA. BALB/c female mice were nasally immunized with vehicle (PBS), PspA (5 µg/mouse), or PspA (5 µg/mouse) in combination with polymeric caffeic acid (pCA) (100 µg/mouse) on days 0, 7, and 14. Seven days after the last immunization, serum samples were collected. The PspA-specific immunoglobulin G antibodies in the samples were evaluated by enzyme-linked immunosorbent assay. Data are obtained from three independent experiments. Vehicle, *n* = 9; PspA, *n* = 12; PspA plus pCA, *n* = 12. Significant differences were calculated using the Kruskal–Wallis test with Dunn’s post hoc test. * *p* < 0.05, ** *p* < 0.01, *** *p* < 0.001.

In a previous study, we have reported that intranasal administration of pCA with ovalbumin (OVA), as a model antigen, enhanced OVA-specific antibody production in the mucosal and systemic compartments [25,26]. In this study, we aimed to develop a nasal vaccine against pneumococcal infection using pCA. First, we examined whether the combined intranasal administration of pCA and PspA, a well-known pneumococcal antigen expressed in all pneumococcal strains, enhanced PspA-specific antibody production in the mucosal and systemic compartments. As shown in Figure 1, intranasal immunization with PspA and pCA markedly increased the production of PspA-specific antibodies in the mucosa. The median endpoint antibody titers for nasal IgA, lung IgA, lung IgG, and vaginal IgA were 551.2, 8.100, 456.4, and 162.2, respectively. In contrast, intranasal vaccination with PBS or PspA alone did not result in PspA-specific IgA and IgG production in the nasal, lung, and vaginal compartments under the same experimental conditions. Moreover, intranasal immunization with PspA and pCA markedly increased the production of PspA-specific antibodies in the serum (median endpoint titer = 537,387) compared to intranasal immunization with PspA alone (median endpoint titer = 2288; Figure 2A).

Next, to evaluate the type of immune response induced by intranasal immunization with PspA and pCA, we examined the production of PspA-specific IgG1 and IgG2a in the serum since the murine IgG subclasses well reflect the helper T (Th) responses to antigens [37]. Figure 2B shows that intranasal immunization with PspA and pCA significantly enhanced the production of PspA-specific IgG1 compared to that of PspA-specific IgG2a, suggesting that pCA exhibits a Th2-biased immune response. Taken together, these results indicate that intranasal immunization with PspA and pCA strongly induces PspA-specific mucosal and systemic antibody responses.

### 3.2. Nasal Vaccination with PspA and pCA Confers Protective Immunity against a Lethal Dose of Pneumococcal Infection

**Figure 3:**
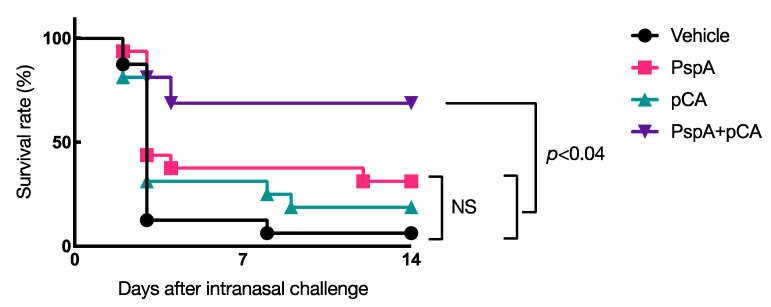
Protective effect of intranasal vaccination with PspA and pCA on the survival rate of mice against pneumococcal infections. BALB/c female mice were nasally immunized with vehicle (PBS), PspA alone (5 µg/mouse), pCA alone (100 µg/mouse), or PspA (5 µg/mouse) in combination with pCA (100 µg/mouse) on days 0, 7, and 14. Seven days after the last immunization, BALB/c female mice were respiratory challenged with *Streptococcus pneumoniae* (5.0 × 10^6^ colony forming units/mice). Survival was monitored for 14 days after challenge. Data were collected from two independent in vivo infection experiments. Vehicle, *n* = 16; PspA alone, *n* = 16; pCA alone, *n* = 16; PspA plus pCA, *n* = 16. Significant differences in the survival rate among the groups were assessed using the Mantel–Cox test. The *p*-values are shown in the figure.

Next, to determine whether intranasal immunization with PspA and pCA induces protective immune responses against *S. pneumoniae* infections, mice were nasally immunized with PBS, PspA (5 µg/mouse) alone, pCA (100 µg/mouse) alone, or PspA (5 µg/mouse) plus pCA (100 µg/mouse) once a week for 3 consecutive weeks. One week after the last immunization, mice were nasally challenged with *S. pneumoniae* (5.0 × 10^6^ CFUs/mouse), and their survival rates were monitored for 14 days. The survival rate after pneumococcal infection was negligible in the group that received intranasal immunization with PBS (<10%) or PspA alone (approximately 30%) (Figure 3). In contrast, >70% mice that were intranasally immunized with PspA in combination with pCA survived under the same experimental conditions (Figure 3). Notably, there was no improvement in the survival rate of mice nasally immunized with pCA alone (Figure 3), implying that the improvement in the survival rate of mice nasally vaccinated with PspA and pCA was mediated via antigen-specific immune responses.

## 4. Discussion

Mucosal vaccines are one of the most promising tools for the prevention of infectious diseases. Vaccines inducing both mucosal and systemic immune responses are considered superior to existing parenteral vaccines as they can effectively prevent pathogens from entering the host during the early stages of infection. This can be achieved by using mucosal vaccine systems. However, it is essential to develop safe mucosal adjuvants to effectively elicit antigen-specific immune responses in the mucosa. In this study, we showed that intranasally administered mucosal vaccine systems, using enzymatic polyphenols in combination with PspA, can elicit antigen-specific immune responses in the mucosal and systemic compartments. Further, the mucosal vaccine system was found to have protective effects in a mouse model of pneumococcal infections. These results indicate that the novel intranasal pneumococcal vaccine developed in this study is promising for controlling pneumococcal infections.

In this study, we have demonstrated that intranasal immunization with pCA and PspA induces mucosal and systemic PspA antigen-specific antibody responses (Figure 1 and Figure 2). Additionally, pCA preferentially elicited a Th2 response as antigen-specific IgG1 production was higher than antigen-specific IgG2a production (Figure 2). A limitation of this study, however, is that we did not evaluate the in vitro production of antigen-specific cytokines from leukocytes, derived from mice intranasally immunized with PspA and pCA, to further investigate the type of immune response elicited by pCA.

Given that this method of immunization could produce PspA-specific antibody responses, we investigated the protective effect of this mucosal vaccine system against pneumococcal infection using in vivo infection experiments. We found that it induced a protective immune response against pneumococcal infection in mice (Figure 3).

Although the detailed mechanism of induction of protective immune response against pneumococcal infection is not fully understood in this study, the fact that intranasal administration of pCA alone did not induce a protective immune response against pneumococcal infection suggests that at least an antigen-specific immune response is responsible for the protective effect against infection. In previous studies, the PspA-specific IgA response was considered to prevent the initial stages of *S. pneumoniae* colony formation and invasion [38]. Moreover, the PspA-specific serum IgG response is responsible for the elimination of invading *S. pneumoniae* from the host [16]. For instance, PspA can immobilize the complement component C3 [39], a humoral arm of innate immunity, to inhibit its deposition, thereby eliminating *S. pneumoniae* from the blood [40]. Therefore, it is likely that PspA-specific serum IgG promotes bacterial elimination from the host.

We could not elucidate the mechanism(s) of mucosal adjuvant effects of pCA using enzymatically synthesized polyphenols as a nasal vaccine system. However, recent studies have indicated that polyphenols can form complexes with peptides and proteins. These complexes are formed by hydrophobic or hydrophilic interactions of polyphenols with proline, phenylalanine, and arginine in the peptides and proteins [41,42]. Hence, we are currently investigating the possibility that polyphenols form nanocomplexes with antigen proteins and function as carriers of antigen delivery, thus eliciting antigen-specific immune responses.

## 5. Conclusions

In conclusion, we developed a novel nasal pneumococcal vaccine system using enzymatically polymerized caffeic acid as a mucosal adjuvant. Our results confirm that enzymatically pCA is a promising safe and effective mucosal adjuvant candidate for vaccination systems to combat airway infections.

## Data Availability

Data is contained within the article or Supplementary Materials.

## Supplementary Materials

The following are available online at https://www.mdpi.com/article/10.3390/pharmaceutics13040585/s1, Figure S1: Preparation of recombinant PspA protein.
