# Supplementary material for: Polymeric Caffeic Acid Acts as a Nasal Vaccine Formulation against Streptococcus pneumoniae Infections in Mice
Source: Pharmaceutics. 2021 Apr 20;13(4):585. doi: 10.3390/pharmaceutics13040585 (PMC8073337; doi:10.3390/pharmaceutics13040585)
Supplement: Supplementary file 1 [file pharmaceutics-13-00585-s001.zip › pharmaceutics-1189507-supplementary.pdf]

pharmaceutics

Supplementary Materials: Polymeric Caffeic Acid Acts as a Nasal Vaccine Formulation against *Streptococcus pneumoniae* Infections in Mice

Rui Tada, Hidehiko Suzuki, Miki Ogasawara, Daisuke Yamanaka, Yoshiyuki Adachi, Jun Kunisawa and Yoichi Negishi

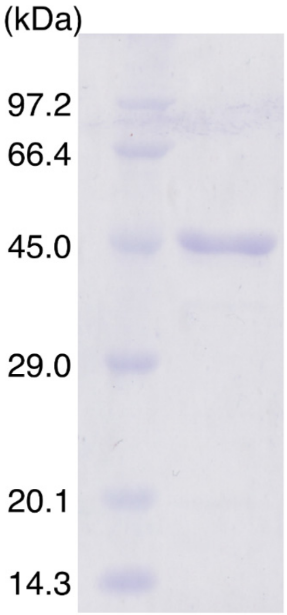

Figure S1. Preparation of recombinant PspA protein. The recombinant PspA protein was subject to SDS-PAGE followed by CBB staining. Lane 1; size marker, Lane 2; PspA.
